# A SOFT DEATH: PERCEPTION AND ATTITUDES TOWARD PALLIATIVE CARE IN SENEGAL

**DOI:** 10.1093/geroni/igae098.1716

**Published:** 2024-12-31

**Authors:** Hannah Silverstein, Fursan Sahawneh, Brian Carpenter

**Affiliations:** Washington University in St. Louis, United States; Rush Medical College, Chicago, Illinois, United States; Washington University in St. Louis, United States

## Abstract

Palliative care (PC) is a specialty medical service that addresses the bio-psycho-social-spiritual needs of patients with serious illnesses and their care partners. Despite the value of PC, its expansion has been underdeveloped in sub-Saharan Africa due to variability in access to training resources and funding. This study sought to understand the current landscape of PC services in Senegal and the barriers and opportunities in its growth. Semi-structured interviews were conducted with six PC clinicians, four caregivers, one driver for a PC service, and one public health professor, all native to Senegal. Questions addressed their experiences delivering or receiving PC and future recommendations. Interviews were translated and transcribed from French into English. Transcripts were qualitatively coded for themes using open and focused coding. Five themes were identified: 1) the current landscape of PC, 2) barriers to implementing PC, 3) strategies and philosophies in care, 4) unique features of Senegalese culture affecting PC, and 5) the future of PC. Our findings demonstrate that PC in Senegal remains an under-resourced and underutilized specialty medical service, but PC is being implemented by personally committed clinicians. Results also highlight the importance of cultural factors influencing care in Senegal and the need to expand training opportunities for clinicians, increase education of other medical providers and the public, integrate PC into the healthcare system, and expand research to evaluate the impact of these resources. PC has the potential to be an important force for improving the quality of life for Senegalese patients and their care partners.

